# Editorial: Porcine reproductive and respiratory syndrome virus—Microbiology and pathogenesis

**DOI:** 10.3389/fmicb.2023.1153923

**Published:** 2023-02-23

**Authors:** Chunhe Guo, Xiaoxiao Zhang, Yongjie Chen, Jiecong Yan, Zhan He

**Affiliations:** ^1^Guangdong Laboratory for Lingnan Modern Agriculture, Guangzhou, Guangdong, China; ^2^Key Laboratory of Zoonosis Prevention and Control of Guangdong Province, College of Veterinary Medicine, South China Agricultural University, Guangzhou, Guangdong, China

**Keywords:** porcine reproductive and respiratory syndrome virus, pathogenesis, interaction, disease, control

Porcine reproductive and respiratory syndrome (PRRS), caused by PRRS virus (PRRSV), is one of the severest infectious diseases in pigs since the late 1980s. This disease is characterized by producing reproductive failures in sows and respiratory problems and growth reduction in pigs of all ages. PRRSV is an enveloped, single-stranded, positive sense RNA virus which is classified as *Betaarterivirus suid* 1 (European type, PRRSV-1) and *Betaarterivirus suid* 2 (American type, PRRSV-2). It is highly host-restricted to cells of the monocyte/macrophage lineage such as porcine alveolar macrophages (PAMs). Since the virus invades immune cells, it destroys the immune system which in turn causes immunosuppression. This helps to develop secondary bacterial infections and interferes with the immune protective effects of other commercial vaccines regarding the pathogens of porcine circovirus and classical swine fever virus. Further, long viremia (>30 days) and antibody-dependent enhancement phenomenon were observed in pigs upon PRRSV infection. At present commercial vaccines (modified live vaccines) are available and valuable but they cannot induce high levels of neutralizing antibodies and thus do not provide effective protection against PRRSV infection. Additionally, these vaccines have others flaws such as long viremia, easy recombination with field strains and so on. Therefore, PRRS vaccine is a double-edged sword and vaccination is highly controversial. To better prevent and control the disease, the pathogenesis of the virus requires further research. The aim of this special issue is to gather a collection of Review or Original Research Articles regarding PRRSV pathogenesis and pathogen-host interactions.

Several potential cellular factors were identified to mediate PRRSV attachment and internalization. Among them, CD163 is an indispensable host factor for the virus entry into target cells both in vivo and in vitro. Functionally, CD163 is well described as a receptor for binding of tumor necrosis factor (TNF)-like weak inducer of apoptosis, pathogen entry into host cells, and hemoglobin clearance through hemoglobin-haptoglobin complexes, which is crucial for physical health. Several cell lines such as PK-15 and BHK-21 transfected with a plasmid expressing porcine CD163 protein confer susceptibility to the infection of PRRSV. Therefore, the CD163 molecule can be used as a key target for PRRS control. Zhang and Guo comprehensively summarized the recent advances in inhibition of PRRSV replication via targeting CD163 receptor. Currently, there are four strategies that can be used to target CD163. (i) Targeting mRNA. MicroRNAs (miRNAs) are capable of binding to CD163 mRNA to induce its degradation, ultimately blocking the translation of CD163 protein.(ii) Blocking binding. In PRRSV life cycle, CD163 interacts with viral envelope proteins such as GP2, GP3, and GP4 to mediate virus uncoating. Thus, small molecules such as peptides and antibodies can interfere with the binding of CD163 to virus proteins to block uncoating ultimately limiting PRRSV replication.(iii) Regulated by its upstream factors or compounds. CD163 is considered to be an anti-inflammatory factor and several factors are reported to have the ability to regulate its expression. It is reported that upstream factors such as TREM2 and ADAM17 can regulate CD163 expression to modulate PRRSV infection. In addition, several compounds that are involved in the regulation of inflammatory cytokine expression are also capable of modulating CD163 expression. (iv) Gene-editing. CRISPR/Cas9-mediated gene modification enables us to edit CD163 to produce genetically modified animals. The glycoproteins of the virus cannot bind to the modified CD163 thereby blocking uncoating and ultimately repressing virus proliferation. Among these approaches, gene-editing is the most promising for breeding CD163-modified pigs against PRRSV infection in clinical application. Since CD163 serves as a scavenger receptor responsible for mediating the removal of hemoglobin from blood plasma upon intravascular hemolysis and pathogens in host innate immune responses. the complete knockout of this gene may have key physiological effects on animal development and growth as well as susceptibility to infection with other pathogens. To preserve the biological functions of CD163 as much as possible, precise mutation of specific amino acids within the scavenger receptor cysteine-rich domain 5 of CD163 may confer resistance of pigs to infection with PRRSV. The molecular mechanisms of PRRSV uncoating mediated by CD163 remain largely unknown. There may be other important cofactors interacting with CD163 to mediate uncoating which can be used as targets for the design of antivirals against PRRSV.

PRRSV has the intrinsic ability to adapt and evolve. PRRSV-2 can be divided into five genetic subtypes in China: lineage 1, 3, 5, 8, and 9. Lineage 1 includes representative strains such as NADC30, NADC34 and RFLP 1-4-4. Zhao et al. wrote a review regarding recent progress in the evolution of NADC34-like strains in China. So far, the strains have spread to ten provinces in China. Therefore, different pathogenicity and morbidity as well as mortality have been developed. Commonly recombination is considered a critical factor for the maintenance of PRRSV strain diversity and recombination of NADC34-like strains increases the complexity of PRRSV virulence and pathogenicity. To prevent and control the emergence of new virulent variants in future, continuous monitoring of the prevalence of NADC34-like strains in pig farms is urgently needed. PRRSV infection leads to immune system dysfunction. T-cell activation requires a proper interaction and precise balance between costimulatory and coinhibitory molecules. Ruedas-Torres et al. reported that a mild increase of costimulatory genes and a stronger up-regulation of coinhibitory genes in targets (especially in lung tissues) of pigs infected with PRRSV-1 were observed, indicating that the expression of coinhibitory genes may be capable of controlling the inflammation-induced body damage synergistically. It remains unknown how the PRRSV spreads in semen and infects boars in the field. Pedersen et al. described the viral distributions and histopathological changes in the reproductive tissues of the infected boars. This study contributes to the establishment of the relationship between findings in boars experimentally and naturally infected with PRRSV.

The control and eradication of PRRS is a worldwide problem. It is critical to address the pathogenesis of the virus and the mechanisms of virus-host interaction, which is a key prerequisite and basis for preventing and controlling it. Since PRRSV infection leads to immunosuppression in pigs, it is difficult to develop ideal vaccines to produce high levels of neutralizing antibodies. Breeding disease-resistant pigs using CRISPR/Cas9 technology and developing antiviral molecules may be important directions for PRRS control.

## Author contributions

All authors listed have made a substantial, direct, and intellectual contribution to the work and approved it for publication.

